# Oral Vaccination with Heat-Inactivated *Mycobacterium bovis* Does Not Interfere with the Antemortem Diagnostic Techniques for Tuberculosis in Goats

**DOI:** 10.3389/fvets.2017.00124

**Published:** 2017-08-07

**Authors:** Alvaro Roy, María A. Risalde, Carmen Casal, Beatriz Romero, Lucía de Juan, Ahmed M. Menshawy, Alberto Díez-Guerrier, Ramon A. Juste, Joseba M. Garrido, Iker A. Sevilla, Christian Gortázar, Lucas Domínguez, Javier Bezos

**Affiliations:** ^1^CZ Veterinaria S.A., Porriño, Pontevedra, Spain; ^2^SaBio, Instituto de Investigación en Recursos Cinegéticos IREC (CSIC-UCLM-JCCM), Ciudad Real, Spain; ^3^VISAVET Health Surveillance Centre, Complutense University of Madrid, Madrid, Spain; ^4^Faculty of Veterinary Medicine, Department of Animal Health, Complutense University of Madrid, Madrid, Spain; ^5^Faculty of Veterinary Medicine, Beni-Suef University, Beni-Suef, Egypt; ^6^MAEVA SERVET S.L., Madrid, Spain; ^7^Servicio Regional de Investigación y Desarrollo Agrario (SERIDA), Villaviciosa, Spain; ^8^Animal Health Department, NEIKER-Tecnalia, Derio, Bizkaia, Spain

**Keywords:** tuberculosis, goat, oral vaccination, heat-inactivated vaccine, diagnosis, interference

## Abstract

Vaccination against tuberculosis (TB) is prohibited in cattle or other species subjected to specific TB eradication campaigns, due to the interference that it may cause with the official diagnostic tests. However, immunization with a heat-inactivated (HI) *Mycobacterium bovis* vaccine *via* the oral route has been suggested to overcome this issue. In this study, the main goal was to assess the interference of the HI vaccine by different routes of administration using a previous vaccination and re-vaccination (boosting) protocol. TB-free kid goats were divided into three groups: oral (*n* = 16), intramuscular (IM; *n* = 16), and control (*n* = 16). Results showed that there was a significant difference in the percentage of animals positive to the single intradermal test (SIT) and blood based interferon-gamma release assay (IGRA) caused by vaccination when performed in the IM group compared to the oral group (*p* < 0.001). Nevertheless, no positivity to the SIT or IGRA test was observed in orally vaccinated goats regardless of the different interpretation criteria applied. None of the groups presented positive antibody titers using an in-house ELISA and samples collected 2 months after the boost. These results suggest the potential usefulness of the HI vaccine by the oral route in goats to minimize the interference on diagnostic tests (skin and IGRA tests) and reducing the necessity of defined antigens to replace the traditional purified protein derivatives for diagnosis. Finally, the results pave the way to future efficacy studies in goats using different routes of HI vaccination.

## Introduction

Tuberculosis (TB) in goats is mostly caused by *Mycobacterium bovis* and *Mycobacterium caprae*, both of which have also been isolated in Spain from other domestic and wildlife species (1–3). According to the Official TB Eradication Program for cattle in Spain, only caprine flocks coexisting, sharing pastures, or having epidemiological links with cattle should be subjected to official tests for the diagnosis of TB. Nevertheless, some regions with a high density of caprine flocks have implemented specific TB control programs for goats (4). These programs are mainly based on the application of skin tests [single intradermal tuberculin test and comparative intradermal tuberculin test, single intradermal test (SIT) and SCIT tests, respectively], while using the blood-based interferon-gamma release assay (IGRA) as an ancillary test under specific circumstances (2). Vaccination against TB is prohibited in cattle in the European Union (Chapter III, Article 13, Council Directive 78/52/EEC). Nevertheless, vaccination has been suggested as a suitable complementary strategy for the control of TB not only in cattle but also in other livestock and wildlife species under certain epidemiological circumstances, and may play a significant role in countries where domestic and wildlife species are not subjected to compulsory TB eradication programs (5). Bacillus Calmette-Guérin (BCG) is the only vaccine approved for use in humans, and it has been assayed in multiple experimental field trials (alone or in combination with others vaccines) in domestic and wild animals, displaying different results in terms of protection conferred (6–12). However, BCG vaccination sensitizes animals to respond to skin and IGRA tests (13), complicating the differentiation between infected and vaccinated animals (14). Therefore, implementation of BCG vaccination as a TB control strategy in livestock would require diagnostic tools able to distinguish vaccinated from infected animals (DIVA) (15, 16) as it was stated in a recent EFSA scientific opinion (17). However, in general terms, diagnostic tools based on DIVA antigens are less sensitive than those using purified protein derivatives (PPDs), increasing the occurrence of false negative reactions (9, 18, 19). Therefore, the development of immunization schemes against TB to reduce the diagnostic interferences and the use of DIVA antigens would be of paramount importance, since they could accelerate the control/eradication process. Such schemes may also be more cost-effective, which is particularly important for other species than cattle.

A heat-inactivated (HI) *M. bovis* vaccine has been recently developed, conferring similar protection to BCG against TB in wild boar (20) and improving the stability under field conditions (21). Both parenteral and oral vaccinations with IV have shown a significant reduction in TB-compatible lesions in wild boar (20, 22). The efficacy of the HI vaccine against a virulent *M. bovis* field strain has been also demonstrated in domestic pigs, reducing significantly the lesion and culture scores (23). In these previous studies, the absence of a sensitization effect was suggested, showing neither IGRA nor ELISA positivity in orally immunized wild boar. By contrast, a consistent response to the IGRA and ELISA test was triggered when animals were vaccinated *via* the IM route (20, 24). Another study confirmed that oral HI vaccine does not sensitize farmed red deer and therefore does not cause false-positive responses in the tuberculin skin test (25). Moreover, a recent study in cattle confirmed these previous findings, demonstrating that the oral vaccination with HI vaccine in calves do not compromise official TB diagnostic tests (26). Nevertheless, the immunization protocol applied in previous studies only considered a single dose and therefore, the effect on the interference of a second boosting re-vaccination, recommended to increase the protection efficacy, was not evaluated. Moreover, evaluation of interference on serological tests may be of interest in goats, since these diagnostic tools are more frequently used in livestock and wildlife not subjected to official eradication programs (27–29).

The aim of the present experiment was to assess the interference caused by the HI vaccine using a vaccination and boosting re-vaccination protocol by two different routes of administration (oral and parenteral) on the cellular and humoral TB diagnostic tests in kid goats. These results will be useful for the design of potential immunization protocols applied in goats with the aim of reducing the high prevalence of caprine TB in regions of Spain where caprine production is uncoupled to the cattle industry and where the TB prevalence is high.

## Materials and Methods

### Animals

Forty-eight female kid goats (2–3 weeks old) were selected from a flock without a previous history of TB. Animals were tested in the farm of origin using a commercial IGRA kit (Bovigam, Thermo Fisher Scientific, USA) and an indirect in-house ELISA that was carried out with a readjusted procedure used by Che-Amat et al. (30) to check their negative status. Afterward, the selected animals were moved to biosafety facilities during the study. Handling of the animals and sampling were performed according to European (Directive 2010/63/EU) and Spanish Legislation (RD 53/2013), and also approved by the Ethics Committee (Complutense University of Madrid) and the Regional Agriculture Authority [Comunidad de Madrid; permit number: PROEX 143/15 (29/06/2015)].

### Experimental Design

Kid goats were randomly distributed into three groups. Group 1 (*n* = 16) non-vaccinated (control); Group 2 (*n* = 16) intramuscularly vaccinated (IM); and Group 3 (*n* = 16) orally vaccinated.*M. bovis* strain Neiker 1403 (spoligotype SB0339) from a naturally infected wild boar was used for the preparation of vaccines as previously described (20). The IM vaccine contained adjuvant Montanide ISA 50 V2 (Seppic, Castres, France), while the oral vaccine consisted of phosphate-buffered saline (PBS) containing the inactivated mycobacteria. Both parenteral and oral inactivated vaccine contained 6 × 10^7^ CFU, and were administered in a 2 mL/dose and 1 mL/dose by oral an IM routes 1 week after arrival, respectively. Groups 2 and 3 received a revaccination 4 weeks after the prime vaccination using the same doses and routes of administration.

### Diagnostic Methods

#### Skin Tests

Both SIT and SCIT tests were carried out 2 months after booster vaccination. SIT and SCIT tests were performed in the neck (cervical region), using PPD of *M. bovis* (bovine PPD) and *M. avium* (avian PPD) (CZ Veterinaria, Spain) according to the R.D. 2611/1996 and European Commission Council Directive 64/432/EEC. The skin fold thickness was measured after 72 h. Bovine and avian PPDs were inoculated on the left and right side of the neck, respectively, with a volume of 0.1 mL using a Dermojet^®^ syringe (Akra Dermojet, France). Applying the standard interpretation of the SIT test, animals were considered as positive reactors if presenting a skin fold thickness increase of 4 or more mm or the presence of clinical signs (exudation, edema, or necrosis). Positive animals to the SCIT test were those showing a positive bovine reaction greater than the avian reaction in more than 4 mm, or the presence of clinical signs at the bovine PPD inoculation site.

#### Interferon-Gamma Release Assay (IGRA)

Blood samples were collected just prior to the vaccination to check the negative status of the goats, and 2 months after re-vaccination. Heparinized whole blood samples were stimulated with bovine and avian PPD (CZ Veterinaria, Spain) at a final concentration of 20 µg/mL and nil antigen PBS as previously described (31–33). Afterward, samples were incubated during 18–20 h at 37° in a humidified atmosphere. The whole blood IGRA was performed following the manufacturer’s instructions (Bovigam^®^ TB Kit, Thermo Fischer Scientific, USA). According to the interpretation criteria prescribed in the Bovine Tuberculosis Spanish Eradication Program, animals were considered positive if the optical density (OD) in the bovine PPD stimulated sample minus the OD of nil antigen sample (PBS) was equal or greater than 0.05 and greater than the avian PPD-stimulated sample. Moreover, a less stringent threshold of 0.1 was also applied for comparison purposes.

#### Serological Analyses

Goats were tested before vaccination and 2 months after booster vaccination in order to evaluate the humoral response against different antigens using an in-house ELISA with some modifications to that previously described by Che-Amat et al. (30). Briefly, testing plates were coated with bovine and avian PPDs, as well as paratuberculosis protoplasmic antigen 3 (PPA3; Allied Monitor, Fayette, MO, USA) at 5 µg/ml in carbonate–bicarbonate buffer (Sigma, Barcelona, Spain), and incubated overnight at 4°C. Following one wash with PBS solution containing 0.05% Tween 20 (PBS-T), wells were blocked with 5% skimmed milk powder solution in PBS (SM) for 60 min at room temperature (RT). Sera were added in duplicate at 1:100 dilution in SM, incubated for 60 min at 37°C, and subsequently washed three times with PBS-T. Horseradish peroxidase-conjugated rabbit anti-sheep IgG antibody (SouthernBiotech, Birmingham, AL, USA) at a dilution of 1:2,000 was added, and the plates were incubated for 30 min at RT and then were washed five times with PBS-T. Color was developed by adding o-phenylenediamine dihydrochloride substrate (FAST OPD, Sigma-Aldrich, St Louis, MO, USA) and incubating the plates for 20 min in darkness and RT conditions. The reaction was stopped with H_2_SO_4_ (3 N), and the OD was measured at 490 nm with an ELISA reader. Goat negative and positive control sera were included in every plate in quadruplicate. Negative control serum was obtained from a TB-free goat previously described as *M. bovis* culture negative from a bTB-free area. Positive control serum was obtained from a goat previously infected with *M. bovis* in an experimental study. Sample results were expressed as an ELISA percentage (*E*%), according to the following formula: [sample *E*% = (mean sample OD/(2 × mean of negative control OD)) × 100]. The cutoff value was calculated using a ROC analysis and was defined as the ratio of the mean sample OD to the double of the mean OD of the negative control. Serum samples with *E*% values greater than 100 were considered as positive.

### Statistical Analysis

Wilson’s 95% confidence intervals (95% CI) were calculated for the percentage of reactors to the different techniques. Comparison of proportions of test reactors between groups was performed using Fisher’s exact test. Skin fold thickness, IFN-γ OD, and ELISA percentage (*E*%) among groups were analyzed by Kruskal–Wallis and Mann–Whitney *U* tests. Agreement between tests was measured with the kappa statistic (*k*) and interpreted as follows: <0.00 poor, 0.00–0.20 slight, 0.21–0.40 fair, 0.41–0.60 moderate, 0.61–0.80 substantial, and 0.81–1.00 almost perfect agreement (34). All previous tests were carried out using SPSS Statistics 20 (IBM, New York, NY, USA), and interpreted considering a *p*-value of 0.05 to determine statistical significance. The normality of the quantitative values was assessed using the Kolmogorov–Smirnov test before further analyses were carried out.

## Results

All animals were negative to the IGRA and the ELISA prior to vaccination. Four animals in the oral group [25.00% (95% CI 10.18–49.50%)], three in the IM vaccinated group [18.75% (95% CI 6.59–43.01%)] and two belonging to the control group [12.50% (95% CI 3.50–36.02%)], presented antibody titers against avian PPD above the cutoff point selected (Table 1).

**Table 1:** Number of positive animals using skin tests, IGRA, and In-house ELISA in the different groups of goats 1 week before vaccination (pre-vaccination analysis) and 8 weeks after booster vaccination (post-vaccination analysis).

Pre-vaccination analysis						Post-vaccination analysis						
Group	IGRA		ELISA			Skin tests		IGRA		ELISA		
	Cutoff 0.05	Cutoff 0.1	PPA3	PPD-A	PPD-B	SIT^a^	SCIT^b^	Cutoff 0.05	Cutoff 0.1	PPA3	PPD-A	PPD-B
Control (*n* = 16)	0 (0.00–19.36%)	0 (0.00–19.36%)	0 (0.00–19.36%)	2/12.50% (35–36.02%)	0 (0.00–19.36%)	0 (0.00–19.36%)^c^	0 (0.00–19.36%)	0 (0.00–19.36%)	0 (0.00–19.36%)	0 (0.00–19.36%)	0 (0.00–19.36%)	0 (0.00–19.36%)
IM (*n* = 16)	0 (0.00–19.36%)	0 (0.00–19.36%)	0 (0.00–19.36%)	3/18.75% (6.59–43.01%)	0 (0.00–19.36%)	11/68.75% (44.40 -85.84%)	2/12.50% (3.5–36.02%)	10/62.50% (38.64–81.52%)	7/43.75% (23.10–66.82%)	0 (0.00–19.36%)	0 (0.00–19.36%)	1/6.25% (1.11–28.33%)
Oral (*n* = 16)	0 (0.00–19.36%)	0 (0.00–19.36%)	0 (0.00–19.36%)	4/25% (10.18–49.50%)	0 (0.00–19.36%)	0 (0.00–19.36%)	0 (0.00–19.36%)	0 (0.00–19.36%)	0 (0.00–19.36%)	0 (0.00–19.36%)	0 (0.00 – 19.36%)	0 (0.00–19.36%)

*^a^Single intradermal tuberculin test (standard interpretation). Positive reactors were considered those showed a skin fold thickness increase of 4 and more mm or the presence of clinical signs (exudation, edema, necrosis)*.

*^b^Single intradermal comparative cervical tuberculin test (standard interpretation). Positive reactors were those showed a bovine reaction of 4 or more mm greater than the avian reaction, or the presence of clinical signs at the bovine PPD inoculation site*.

*^c^Wilson’s 95% confidence intervals*.

*PPA3, paratuberculosis protoplasmic antigen 3; PPD-A, avian tuberculin purified protein derivative; PPD-B, bovine tuberculin purified protein derivative*.

**Figure 1:**
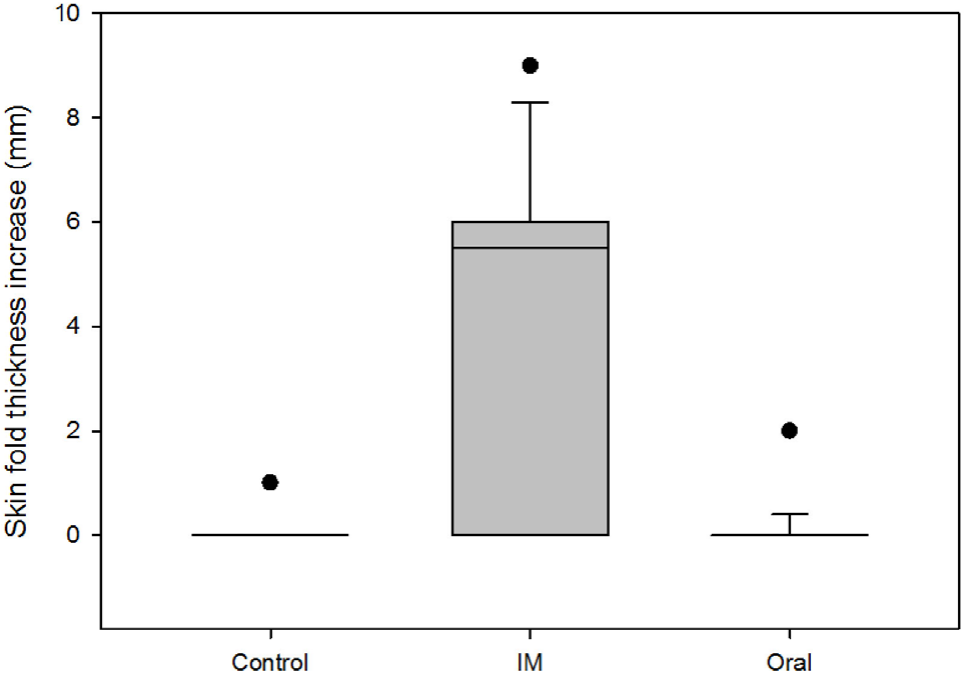
Comparison of the skin fold thickness increase (mm) at the bovine purified protein derivative (PPD) site among groups 2 months after booster vaccination. Boxes represent the lower, median, and higher quartile ranges and “outliers” are represented by closed circles.

Regarding to the SIT and SCIT tests 2 months after booster immunization, 68.75% (95% CI 44.40–85.84%) and 12.50% (95% CI 3.50–36.02%) of the animals were positive reactors to both tests in the IM vaccinated group, respectively. None of the animals in the control and oral vaccinated groups showed a positive reaction to SIT and SICT tests (Table 1). The skin fold thickness increase in the bovine PPD site was significantly higher in the IM vaccinated group [median 5.5 mm, interquartile range (IQR) 0–6 mm] compared to oral vaccinated (median 0 mm, IQR 0–0 mm; Mann–Whitney *U* test, *p* < 0.001) and to control goats (median 0 mm, IQR 0–0 mm; Mann–Whitney *U* test, *p* < 0.001) (Figure 1). One animal in the IM vaccinated group, presented an increase higher than 4 mm in the avian PPD site. Oral and control groups did not show statistically significant differences in the skin fold thickness increase at the bovine PPD injection site (Figure 1).

**Figure 2:**
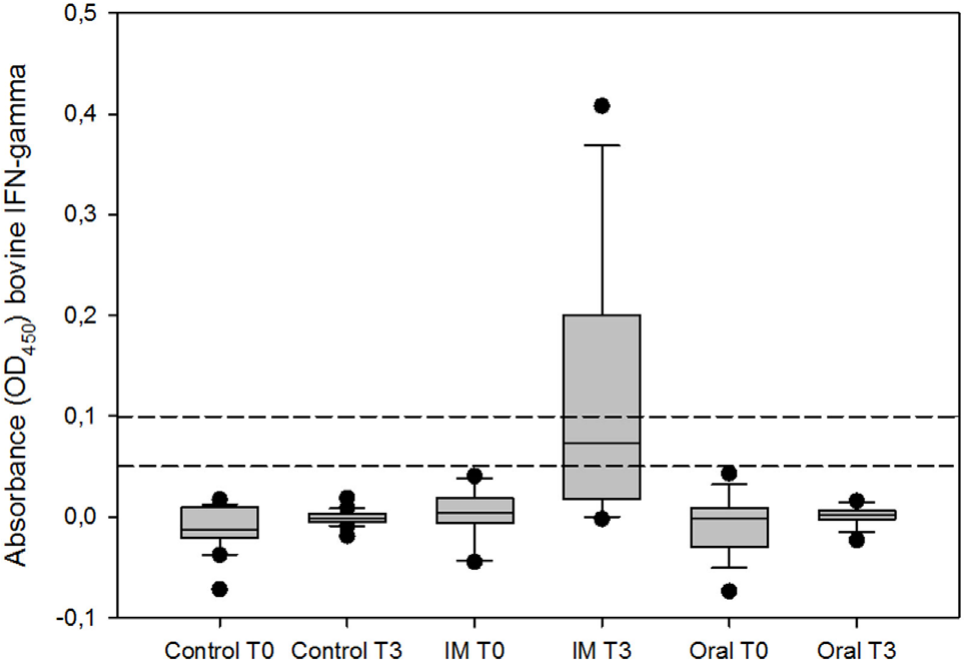
IFN-γ response of whole blood stimulated with bovine purified protein derivative (PPD), expressed as optical density (OD_450_). T0 represents the day of prime vaccination and T3 3 months later. Boxes represent the lower, median, and higher quartile ranges and “outliers” are represented by closed circles. The dashed lines represent the cutoff values used in this study (0.05 and 0.1).

Similar findings were observed using the IGRA, since no positive animals were detected in the oral vaccinated and control group regardless the cutoff point selected (0.05 and 0.1). However, in the IM vaccinated group, there were 10 [62.50% (95% CI 38.64–81.52%)] and 7 [43.75% (95% CI 23.10–66.82%)] positive reactors out of the 16 animals, employing the two cutoff points 0.05 and 0.1, respectively. The percentage of reactors in the IM vaccinated group was significantly higher compared to the other two groups (Fisher’s exact test, *p* < 0.001 for both comparisons). According to these results, significant differences (Kruskal–Wallis test, *p* < 0.001) were found between the optical densities against bovine PPD in the IM vaccinated and the other groups, 2 months after booster vaccination (Figure 2).

Humoral response was measured by an indirect in-house ELISA, detecting only one positive animal to the bovine PPD in the IM vaccinated group 2 months after revaccination. Regarding the oral and control groups, all animals were negative to the bovine PPD, avian PPD, and PPA3. No significant differences (Kruskal–Wallis test, *p* = 0.35) were found in the bovine PPD (*E*%) values among groups 2 months after booster vaccination (Control: median 35.1, IQR 29.9–37.3; IM: median 44.7, IQR 37.9–58.0; Oral: median 38, IQR 29.9–48.4).

The agreement achieved between the SIT test and the whole blood IGRA (cut off 0.05) in all groups was substantial (*k* = 0.695). Nevertheless, no agreement was observed between the ELISA and the cell-based diagnostic techniques (*k* = −0.039, IGRA vs ELISA; *k* = −0.040, SIT vs ELISA; *k* = −0.029, SCIT vs ELISA—Table 2).

**Table 2:** Agreement between tests in all animals 2 months after booster vaccination (kappa index, *k*).

Test	Agreement	Interpretation
Single intradermal test (SIT) vs SCIT	0.255	Slight agreement
SIT vs IGRA (cutoff 0.05)	0.695	Substantial agreement
SIT vs IGRA (cutoff 0.1)	0.459	Moderate agreement
SIT vs ELISA	−0.040	Poor agreement
SCIT vs IGRA (cutoff 0.05)	0.284	Slight agreement
SCIT vs IGRA (cutoff 0.1)	0.406	Moderate agreement
SCIT vs ELISA	−0.029	Poor agreement
IGRA (cutoff 0.05) vs IGRA (cutoff 0.1)	0.787	Substantial agreement
IGRA (cutoff 0.05) vs ELISA	−0.039	Poor agreement
IGRA (cutoff 0.1) vs ELISA	−0.038	Poor agreement

## Discussion

The development of new vaccines may improve control strategies for TB in livestock and wildlife, thereby facilitating the eradication of TB (35, 36). Thus, the application of non-sensitizing vaccines could be a suitable choice, particularly in species not subjected to eradication programs or in regions/countries where the investment in DIVA antigens is not affordable, since the implementation of DIVA tests at a large scale entails high logistical demands and costs.

To the best of our knowledge, this is the first study evaluating the interference on TB cell and antibody-based diagnosis caused by the vaccination with a *M. bovis* HI vaccine in goats, as well as the effect of booster re-vaccination protocols. The present study shows significant differences in the results of the cell-based diagnostic techniques depending on the route of vaccination used. The interference caused on the tuberculin skin tests in the animals vaccinated *via* parenteral 2 months after booster vaccination, yielded a 68.75% and a 12.50% of positive reactors using the SIT and SCIT tests, respectively, while no positive skin test reactions were observed in any of the kids orally vaccinated. These results are in agreement with those reported previously in cattle (26) and red deer (25), where oral vaccination did not cause false-positive responses in the tuberculin skin test. Despite this interference on the skin test caused by an HI vaccine has not been previously described using the parenteral route in goats, there are other studies performed with live vaccines which corroborate these findings (37, 38). Using the IGRA, a high proportion of reactors among intramuscularly vaccinated goats (above 40%) were observed 2 months after booster vaccination, regardless the cutoff point used. These findings are similar to those reported in previous studies in wild boar (20) and cattle (26), where the IM route showed a clear and consistent bovine PPD IFN-γ response, but no positive reactors were detected *via* the oral route. IGRA positive responses to defined antigenic peptide cocktails have been reported following IM vaccination in cattle, whereas no interference was observed *via* the oral route (26). These peptide cocktails could be considered an extra diagnostic tool combining them with oral vaccination, but nowadays, the implementation of these DIVA reagents in goats is not affordable for widespread use.

The lack of positive reactor animals in all groups after vaccination using our in-house ELISA (only one goat vaccinated *via* parenteral showed a positive antibody titer) suggests that animals have not developed a humoral response yet. Long-term immunogenicity studies would be required to evaluate the humoral response elicited by the HI vaccine, administered orally and intramuscularly. Goats vaccinated with subcutaneous BCG and boosting with a recombinant adenoviral vaccine 8 weeks later induce a peak in the antibody response 2 weeks after the boost (10). However, the use of an HI vaccine instead of a live-attenuated vaccine in our study might have delayed this peak, being necessary extended serological studies.

This study demonstrates that the use of oral HI vaccine does not produce interference in diagnostic techniques used in TB eradication programs, as previously was observed in wild boar (20), pig (23), red deer (25), and cattle (26). The mechanism for this absence of interference has not been elucidated although it may be related to the different absorption routes or to the adjuvants used. However, in the present study, several key points must be taken into account comparing with previous studies with the *M. bovis* HI vaccine. First, prime and booster doses of HI vaccine were administered 1 month apart. By contrast, only a single dose was applied in the studies in wild boar, red deer, and cattle (20, 25, 26). Second, animals were only tested 2 months after the booster vaccination, considering sufficient time to induce cell-mediated immunity. Hence, further studies at extended time points are necessary to evaluate the cell mediated immunity induced by the oral HI vaccine throughout time, which could be delayed as described previously in orally BCG vaccinated cattle (39). This limited sampling time schedule was due to these animals entering a second experimental phase where they were subsequently exposed to a group of TB-infected animals in order to continue with an efficacy study. Third, the age of vaccination with the HI vaccine differed among previous studies: red deer (adults), cattle (5–7 months), wild boar (3–4 months), and pigs (3–4 months); sampling schedule after vaccination was also different in these studies: red deer (months 7 and 12), cattle (weekly up to 8 weeks after vaccination), wild boar (day 60), and pigs (day 57) (20, 23–26). Few studies in humans have focused on the role of vaccine administration-related parameters on vaccine efficacy, since factors as the number and interval between immunizations could trigger different immunological responses (40). Moreover, many clinical studies in humans demonstrated that longer intervals between two immunizations may help to achieve better immune responses (41–43). In this sense, we chose an earlier age of vaccination for this study, in concordance with that demonstrated in epidemiological studies in which the beneficial effects of BCG vaccination are greater in very young individuals (44, 45). Moreover, different researchers have highlighted the protection against TB in pigs and wild boar vaccinated orally with HI vaccine suggesting the stimulation of the mucosal immune system (20, 23) and that permeability of the gastrointestinal tract decreases with age (46, 47).

A further advantage of using an inactivated vaccine is that the environmental risks, production costs, and restrictions on storage conditions could be reduced. Therefore, this oral HI vaccine is suitable for its use in wild animals where it is difficult to implement control strategies based on slaughter positive animals to the diagnostic tests (22, 25). Data obtained in goats as experimental models can lay the ground for further studies in large ruminants or humans (48). These considerations might encourage the use of vaccination as an alternative strategy in developing countries, where control strategies based on diagnosis and cull of positive animals are not feasible, or in countries where prevalence of bovine TB is very high (37, 49). The absence of interference caused by the oral HI vaccine in the diagnostic TB tests allows the possibility of dispensing with the implementation of DIVA tests, thereby reducing implementation costs.

In summary, oral vaccination with HI vaccine in kid goats proves to be a suitable route, not causing any interference on TB cell and antibody-based diagnostic techniques, even if applying a booster protocol which could trigger a stronger immune response than a single dose. In addition, this study paves the way for vaccine efficacy studies of HI vaccine in goats. These future efficacy studies could demonstrate the efficacy previously suggested in orally vaccinated wild boars and pigs.

## Ethics Statement

Handling of the animals and sampling were performed according to European (Council Directive 2010/63/EU) and Spanish Legislation (RD 53/2013), and also approved by the Ethics Committee (Complutense University of Madrid) and the Regional Agriculture Authority [Comunidad de Madrid; permit number: PROEX 143/15 (29/06/2015)].

## Author Contributions

Participated in experimental design: AR, MR, CC, BR, LJ, AD-G, RJ, CG, LD, and JB. Prepared the inactivated vaccines: JG and IS. Conducted field and laboratory work: AR, MR, CC, ASM, AD-G, and JB. Performed data analysis: AR, LD, CG, and JB. Drafted the manuscript: AR and JB. Revised the manuscript: MR, RJ, LD, CG, and JB.

## Conflict of Interest Statement

The authors declare that the research was conducted in the absence of any commercial or financial relationships that could be construed as a potential conflict of interest.
